# MicroRNA-142 regulates inflammation and T cell differentiation in an animal model of multiple sclerosis

**DOI:** 10.1186/s12974-017-0832-7

**Published:** 2017-03-16

**Authors:** Farideh Talebi, Samira Ghorbani, Wing Fuk Chan, Roobina Boghozian, Farimah Masoumi, Sedigheh Ghasemi, Mohammed Vojgani, Christopher Power, Farshid Noorbakhsh

**Affiliations:** 1 Department of Immunology, School of Medicine, Tehran University of Medical Sciences, Tehran, Iran; 2 Shefa Neuroscience Research Institute, Khatam Al-Anbia Hospital, Tehran, Iran; 3 Department of Medicine (Neurology), University of Alberta, Edmonton, AB Canada; 4 Multiple Sclerosis Centre, University of Alberta, Edmonton, AB Canada

**Keywords:** MicroRNA, Neuroinflammation, Multiple sclerosis, Experimental autoimmune encephalomyelitis, T cell differentiation

## Abstract

**Background:**

MicroRNAs have emerged as an important class of modulators of gene expression. These molecules influence protein synthesis through translational repression or degradation of mRNA transcripts. Herein, we investigated the potential role of miR-142a isoforms, miR-142a-3p and miR-142a-5p, in the context of autoimmune neuroinflammation.

**Methods:**

The expression levels of two mature isoforms of miR-142 were measured in the brains of patients with multiple sclerosis (MS) and the CNS tissues from mice with experimental autoimmune encephalomyelitis (EAE), an animal model of MS. Expression analyses were also performed in mitogen and antigen-stimulated splenocytes, as well as macrophages and astrocytes using real-time RT-PCR. The role of the mature miRNAs was then investigated in T cell differentiation by transfection of CD4^+^ T cells, followed by flow cytometric analysis of intracellular cytokines. Luciferase assays using vectors containing the 3′UTR of predicted targets were performed to confirm the interaction of miRNA sequences with transcripts. Expression of targets were then analyzed in activated splenocytes and MS/EAE tissues.

**Results:**

Expression of miR-142-5p was significantly increased in the frontal white matter from MS patients compared with white matter from non-MS controls. Likewise, expression levels of miR-142a-5p and miR-142a-3p showed significant upregulation in the spinal cords of EAE mice at days 15 and 25 post disease induction. Splenocytes stimulated with myelin oligodendrocyte glycoprotein (MOG) peptide or anti-CD3/anti-CD28 antibodies showed upregulation of miR-142a-5p and miR-142a-3p isoforms, whereas stimulated bone marrow-derived macrophages and primary astrocytes did not show any significant changes in miRNA expression levels. miR-142a-5p overexpression in activated lymphocytes shifted the pattern of T cell differentiation towards Th1 cells. Luciferase assays revealed SOCS1 and TGFBR1 as direct targets of miR-142a-5p and miR-142a-3p, respectively, and overexpression of miRNA mimic sequences suppressed the expression of these target transcripts in lymphocytes. SOCS1 levels were also diminished in MS white matter and EAE spinal cords.

**Conclusions:**

Our findings suggest that increased expression of miR-142 isoforms might be involved in the pathogenesis of autoimmune neuroinflammation by influencing T cell differentiation, and this effect could be mediated by interaction of miR-142 isoforms with SOCS1 and TGFBR-1 transcripts.

**Electronic supplementary material:**

The online version of this article (doi:10.1186/s12974-017-0832-7) contains supplementary material, which is available to authorized users.

## Background

MicroRNAs (miRNAs) are a family of small noncoding RNAs that function as negative regulators of gene expression through sequence-specific binding to the 3′-untranslated region (3′-UTR) of their target messenger RNAs (mRNAs) [1, 2]. Numerous studies have demonstrated the role of miRNAs in different cell biological processes including cell proliferation, differentiation, apoptosis, and migration by targeting and downregulating the expression of various protein-coding genes [3]. Moreover, dysregulation of specific miRNAs have been associated with human disease, including cancers, infectious diseases, and inflammatory and immune-related disorders. In the context of immunological disorders, miRNAs have been shown to influence the activity and function of both innate and adaptive arms of the immune system [4], which makes them important pathogenic players as well as potential therapeutic targets in these disorders. Autoimmune diseases have been of particular interest to miRNA researchers over the last decade and various miRNAs have been shown to exert critical effects in major autoimmune disorders including diabetes, rheumatoid arthritis, systemic lupus erythematosus, and multiple sclerosis (MS) [5]. In the case of MS, studies on peripheral blood leukocytes and brain tissue have shown altered expression of various miRNAs [6–8]. In one of the first studies performed on peripheral blood leukocytes from MS patients, Otaegui et al. reported an association between miR-18b and miR-599 with disease relapses and miR-96 with remissions [6]. In another miRNA study on blood cells, Keller et al. identified a set of 48 miRNAs which could differentiate relapsing-remitting multiple sclerosis (RR-MS) patients from healthy controls with high specificity and sensitivity [9]. Studies focusing on miRNA expression in T cells have revealed altered expression of miRNAs which target genes with known role in T cell activation [10–12]. In addition to association with disease, studies have also shown direct involvement of miRNAs in MS disease pathogenesis. In a seminal work by Du et al., miR-326 was reported to regulate the differentiation of T cell towards the pathogenic Th17 phenotype in MS patients and experimental autoimmune encephalomyelitis (EAE) mice [13]. Investigating the expression of miRNAs in autopsy brain tissue derived from MS patients also supports the role of these molecules in disease process. miRNA profiling on active and inactive brain lesion by Junker et al. has revealed dysregulation of multiple miRNAs including miR-34a, miR-155, and miR-326, which target CD47 regulatory protein, in MS brains [14]. Other miRNA-profiling studies on brain tissue derived from MS patients have also shown dysregulation of multiple miRNA species in MS brain including normal-appearing white matter [15]. In addition to their role in pathogenesis, miRNAs might also be important as therapeutic targets in MS. In the study by Du et al., in vivo silencing of miR-326 resulted in fewer Th17 cells and milder EAE, and its overexpression led to more severe EAE disease [13]. Likewise, treatment of EAE mice with anti-miR-155 sequences have been reported to decrease the clinical severity of EAE, a finding which is consistent with the role of miR-155 in development of Th1 and Th17 cells [16].

In the current study, we focused on the role of miR-142 in autoimmune neuroinflammation that takes place in MS and the EAE. mir-142 is broadly conserved between different species, including human and mouse (Additional file 1: Figure S1). Immature mir-142 generates two mature isoforms; miR-142-3p and miR-142-5p which have both been implicated in regulation of leukocyte activity and also in inflammatory diseases [17–20]. In the context of autoimmune neuroinflammation, upregulation of miR-142-5p have been reported in MS brain tissue in miRNA-profiling studies [5, 8, 14]. Moreover, high expression of miR-142-3P in EAE brain tissue and CSF of patients with multiple sclerosis during active inflammation has been illustrated [21]. Nonetheless, the potential pathogenic or protective role that this miRNA might have in disease process is not fully known.

In this study, we first used human brain autopsy samples as well as central nervous system (CNS) tissue derived from EAE animals at different time points after disease induction to investigate the expression of miR-142-5p and miR-142-3p isoforms in disease tissues. Expression of miRNA isoforms were next measured in cultures of cells with potential roles in MS/EAE pathogenesis. Overexpression experiments in CD4^+^ T cells were performed to examine the effect of miR-142 on T cell differentiation. 3′UTR cloning and luciferase assays were then carried out to identify direct mRNA targets of miR-142, followed by quantifying the expression of targets in CNS tissue and cultured cells.

## Methods

### Human brain studies

The use of autopsied brain tissues was approved by the University of Alberta Human Research Ethics Board (Biomedical, protocol number 2291), and written informed consent was obtained for all samples collected from age- and sex-matched subjects including non-MS patients (*n* = 6; mean age = 61 + 4.0 years; male:female, 3:3; diagnoses at death: sepsis, cancer, myocardial infarction, stroke, HIV-AIDS, Parkinson’s disease) and patients with MS (*n* = 6; mean age = 56 + 3.2 years; male:female, 2:4; diagnoses at death: secondary progressive MS (*n* = 4), primary progressive MS (*n* = 1) and relapsing-remitting MS (*n* = 1). All tissue samples were stored at −80 °C as previously reported [22, 23]. In order to detect demyelinated lesions, luxol fast blue (LFB) staining was performed on brain sections and parts of the tissues which did not show evident demyelination were used for RNA preparation.

### Mice and EAE induction

C57BL/6 wild-type (WT) female mice (8 weeks old) were purchased from The Pasteur Institute of Iran and maintained in the animal facility of Tehran University of Medical Sciences. After 4 weeks, EAE was induced in 12-week-old mice by using MOG35-55 peptide. While both recombinant myelin oligodendrocyte glycoprotein (MOG) protein and MOG35-55 have been used for EAE induction in C57BL/6 mice, in this study, MOG35-55 was used considering its role in inducing EAE by stimulating neuroantigen-reactive T cells [24, 25]. MOG35-55 peptide emulsified in complete Freund’s adjuvant (CFA) was injected subcutaneously at two sites on the back (0.1 ml of emulsion/site) (EK-2110, Hooke Kit™ MOG35-55/CFA Emulsion PTX). On the same day, and on the following day, mice received intraperitoneal injections of pertussis toxin in PBS, at 200 ng/mouse/dose (0.1 ml). Control mice received subcutaneous CFA and intraperitoneal pertussis toxin injections with the same dose as the EAE mice. Animals were assessed daily for disease severity for up to 30 days following immunization using a 0–15-point scoring scale [15]. All experiments conformed to guidelines from the Research Ethics Committee of Tehran University of Medical Sciences. CNS tissues were removed from EAE and control mice at three different time points after disease induction (pre-onset, peak of disease, and post peak phase) and were stored at −80 freezers. Previous analyses of CNS tissue in this model of EAE have revealed that lumbar spinal cord is the location which shows higher levels of inflammation and demyelination more consistently [26, 27]. Hence, in this study, we focused on lumbar spinal cord tissue for further expression analysis.

### Immunohistochemistry

To detect T cell infiltration and demyelination in EAE spinal cords, immunohistochemical staining for CD3 T cell marker and myelin basic protein (MBP) was performed on lumbar spinal cord sections, as previously described [22]. Briefly, formalin-fixed paraffin-embedded spinal cord sections were deparaffinized in xylene and rehydrated in decreasing concentrations of ethanol. Antigen retrieval was performed by boiling the sections in 0.01 M trisodium citrate buffer (pH = 6). Sections were next blocked in 10% normal goat serum containing 0.1% triton X-100 and then incubated overnight at 4° with antibodies against CD3 (1:100; Santa Cruz Biotechnology Inc.) and myelin basic protein (1:500; Sternberger Monoclonal) followed by washing. Sections were then incubated with HRP-conjugated secondary antibodies (1:500, Abcam) followed by color development using DAB substrate solution.

### Splenocytes culture and treatment

Spleens were removed from MOG-immunized mice 7 days after EAE induction; tissues were homogenized; and splenocytes were isolated using Ficoll centrifugation (Inno-Train). 2 × 10^6^ cells were cultured in the presence or absence of different concentrations of MOG-35-55 (MOG_35-55_, Hooke labs) in 24-well plates in a final volume of 1 ml RPMI 1640 medium supplemented with 5% FBS. Treated cells were harvested after 12, 24, and 48 h of incubation at 37 °C. Mouse splenocytes were also cultured in 24-well plates and treated with mouse anti-CD3 (0.5 μg/ml) and anti-CD28 (0.2 μg/ ml) (eBioscience) for different durations from 1 to 72 h at 37 °C in a humidified CO_2_ incubator.

### Macrophage and astrocyte cell cultures and treatment

Bone marrow-derived macrophages and primary mouse astrocyte cultures were prepared, as previously described [28]. Briefly, femur and tibia were removed from euthanized C57BL/6 mice under sterile conditions. The two ends of bones were cut, and bone marrow was expelled with a syringe filled with culture medium. Cells from bone marrow were cultured for 7 days in the presence of 50 ng/ml recombinant macrophage colony-stimulating factor (M-CSF) (eBioscience) [28]. Differentiated macrophages were treated with lipopolysaccharide (LPS) (10 and 100 ng/ml) for 12 h at 37 °C before RNA extraction. For astrocyte cultures, neonatal mouse brain tissue was used. Brains were removed and placed in DMEM medium under sterile conditions. Brain tissues were dissected, and astrocyte cells were cultured in DMEM medium supplemented with 20% FBS. Astrocytes were stimulated with 10 and 100 ng/ml LPS (Sigma Aldrich) for 12 h at 37 °C [29]. To confirm the identity of the cells, we performed immunofluorescent staining using an anti-GFAP antibody (1:250, mouse polyclonal, Abcam).

### RNA extraction and cDNA synthesis

Total RNA, containing microRNAs, was extracted from human brain tissue samples, EAE lumbar spinal cord tissues, stimulated splenocytes, cultured macrophages, and astrocytes using miRNeasy Mini Kit (Qiagen). RNA concentration was determined with a Nanodrop. First-strand cDNA synthesis was performed from 1 μg total RNA using miScript II RT Kit (Qiagen) for microRNA analyses and TAKARA kit for gene expression analyses, according to the manufacturers’ instructions.

### Real-time RT-PCR

MicroRNAs (miR-142-3p and miR-142-5p) and their predicted target levels were measured by real-time reverse transcription–PCR using SYBR Green dye on a Bio-Rad CFX96 system in cells and tissues. MicroRNA expression data were normalized against snord 68 and snord 72 expression levels (Qiagen). Expression of the other genes were normalized against β-actin mRNA levels. Primer sequences used for mRNA expression analysis are shown in Additional file 1: Table S1.

### Cell transfections with miRNA mimics

To analyze the effect of microRNA overexpression on endogenous levels of TGFBR-1, TGFBR-2, and SOCS-1 in cells, mouse splenocyte were transfected with miR-142a-3p and miR-142a-5p mimic sequences at 50nM/ml (Qiagen) using Hiperfect transfection reagent (Qiagen) according to the manufacturer’s protocol. After 4 h, the transfected cells were treated with anti-CD3 (0.5 μg/ml) and anti-CD28 (0.2 μg/ml) (eBioscience) for 48 h at 37 °C. For transfection experiments, AllStars negative control siRNA sequence (Qiagen) was used as a control. Total RNA was extracted from transfected cells using miRNeasy Mini Kit (Qiagen), and the levels of predicted gene targets expression were measured by real-time RT–PCR.

### Luciferase assays and miRNA target verification analyses

In order to verify the interaction of TGFBR1 transcripts with miR-142a-3p as well as TGFBR2 and suppressor of cytokine signaling 1 (SOCS1) transcripts with miR-142a-5p, we used luciferase-3′-UTR reporter system. TGFBR-1, TGFBR-2, and SOCS-1 3′ UTR entire fragments were cloned downstream of the Renilla luciferase coding sequence (NotI/XhoI sites) in the psiCheck-2 plasmid (Promega). 25 × 10^3^ HEK293T cells were cultured in each well of 96-well plates, and the reporter plasmids psiCHECK 3′ UTR (100 ng) were co-transfected along with miR-142a-3p and miR-142a-5p mimic sequences (50 ng) into cultured cells using Attractene transfection reagent (Qiagen) according to the manufacturer’s protocol. For each gene relevant psiCHECK 3′ UTR plus negative control siRNA (Qiagen), co-transfection was used as a control. Following 48 h of incubation at 37 °C, cells were harvested and both Firefly and Renilla luciferase activity were measured using the Dual-Glo dual luciferase assay system (Promega) according to the manufacturer’s protocols. Firefly luciferase activity was normalized to Renilla luciferase expression for each sample [15].

### T cell differentiation

Mouse naive CD4^+^ T cells were isolated from C57/BL6 mice spleens using Ficoll followed by naïve CD4^+^ T cell isolation by negative selection kit (mouse CD4^+^ T cell isolation kit, Miltenyi Biotec). 1 × 10^5^ cells were cultured in each well of 96-well plates and were then transfected with miR-142a-3p and miR-142a-5p mimic 50nM/ml (Qiagen) using Hiperfect transfection reagent (Qiagen) according to the manufacturer’s protocol. After 4 h, the transfected cells were transferred to anti-CD3-coated wells (1 μg/ml) and were treated with soluble anti-CD28 (0.2 μg/ml) (eBioscience). AllStars negative control siRNA sequence (Qiagen) was used as a control. Transfected cells were differentiated to three subtypes of T cells, i.e., T regulatory cells, Th1, and Th17, using three different cytokine regimens. For regulatory T (Treg) cells, transfected cells were cultured in complete RPMI, plate-bound CD3 antibody, and soluble CD28 antibody (0.2 μg/ml), IL-2 (20 ng/ml), and TGF-β1 (50 ng/ml) (BioLegend) for 96 h. For Th1 cells, transfected cells were cultured in complete RPMI, plate-bound CD3 antibody, and soluble CD28 antibody (0.2 μg/ml), IL-2 (20 ng/ml), IL-12 (50 ng/ml), and anti-IL-4 antibody (10 ng/ml) (BioLegend) for 96 h. To differentiate the cells into Th17 cells, transfected cells were cultured in complete RPMI, plate-bound CD3 antibody, and soluble CD28 antibody (0.2 μg/ml), TGF-β (5 ng/ml), IL-6 (100 ng/ml), anti-IFN-γ (10 ng/ml), anti-IL-4 (10 ng/ml), and IL-23 (50 ng/ml) (BioLegend) for 96 h.

### Intracellular staining and flow cytometry

To detect intracellular expression of interferon (IFN)-γ, interleukin (IL)-17A, and FoxP3 in transfected CD4^+^ T cells, cells were surface-stained with anti-CD4 and anti-CD3 antibodies and then fixed with 1 ml/tube BioLegend’s Fixation Buffer, at room temperature in the dark for 20 min. Cells were permeabilized with 1 ml BioLegend’s Permeabilization Buffer (1×) and then stained with flurochrome-conjugated anti- IFN-γ, IL-17A, and Foxp3 antibodies (Biolegend). Stained cells were assayed with a BD FACSCalibur flow cytometer, and results were analyzed with FlowJo software. APC Rat IgG2b, κ Isotype Ctrl antibody, PerCP Rat IgG2b, κ Isotype Ctrl antibody, PE Rat IgG1, and κ Isotype Ctrl antibody were used as isotype controls.

### Statistical analysis

Statistical analyses were performed using SPSS software, Version 20. Student’s *t* and Mann–Whitney *U* tests were used for parametric and non-parametric mean comparisons between the two groups. One-way ANOVA or Kruskal–Wallis tests were performed for parametric and non-parametric mean comparisons between multiple groups. Data are shown as mean + SEM.

## Results

### miR-142 isoforms are upregulated in the CNS of MS patients and animals with EAE

**Fig. 1:**
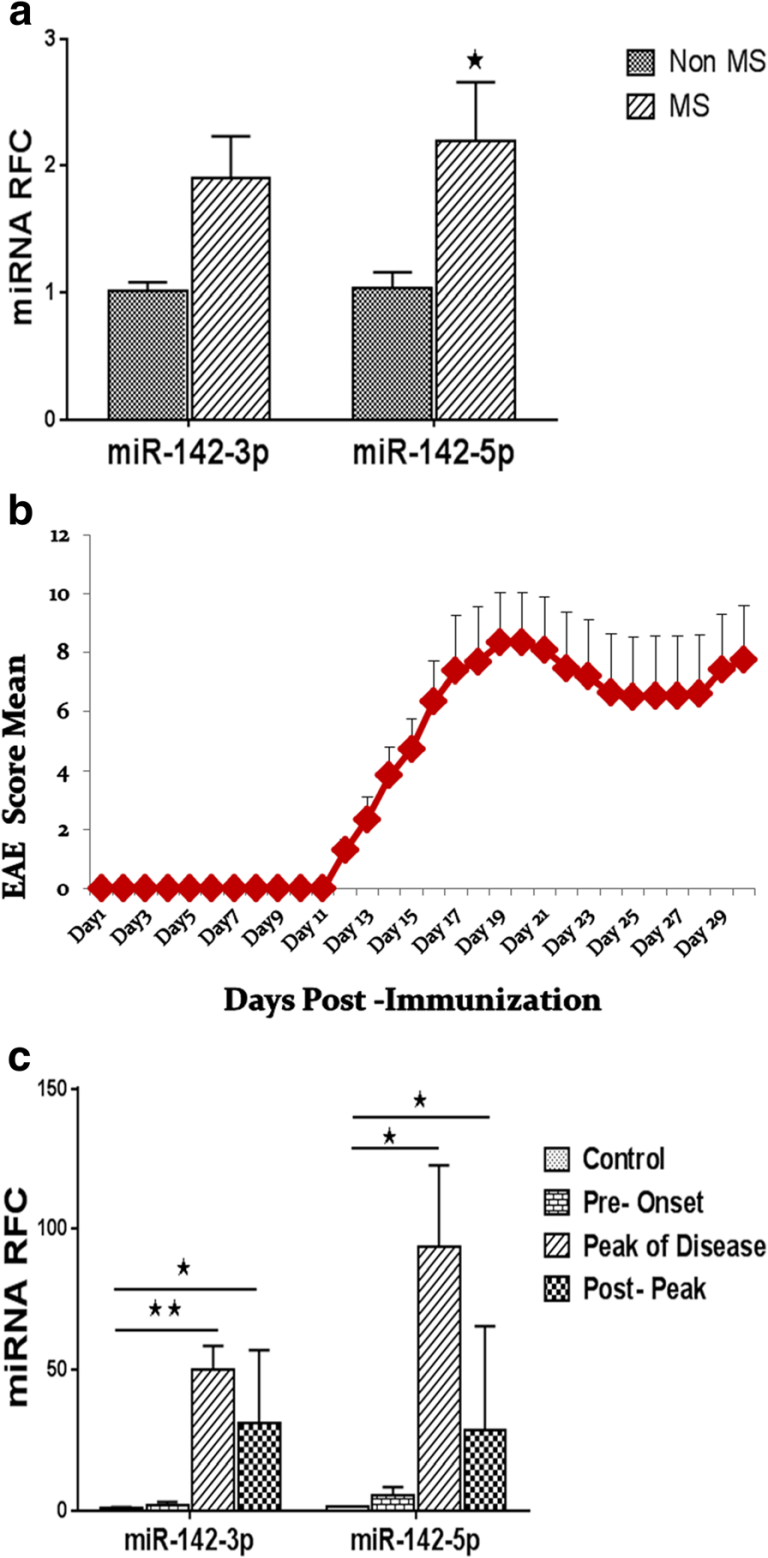
miR-142-3p and miR-142-5p levels in human brain tissue samples and EAE spinal cords. Expression of microRNAs was measured in CNS tissues by real-time RT-PCR. The level of miR-142-5p was significantly increased in human MS samples compared with non-MS controls (**a**) (*n* = 6, Mann–Whitney *U* test, *p ≤ 0.05). EAE was induced in C57BL6 mice and spinal cord tissues were extracted at three time points after disease induction (**b**). Expression levels of miR-142a-3p and miR-142a-5p were significantly increased in spinal cord during the peak of disease and post peak phases of EAE (**c**). Data are shown as mean ± SEM. Number of mice in each group = 10, *p < 0.05, **p < 0.01, Kruskal–Wallis tests

To confirm altered expression of miR-142 in MS white matter, we analyzed the expression of miR-142-3p and miR-142-5p isoforms in normal-appearing cerebral white matter from MS and non-MS cases by real-time PCR. These studies showed that miR-142-5p expression levels were significantly increased in MS brains compared with non-MS brain tissues (Fig. 1a), as previously reported in miRNA-profiling studies [2, 14, 15]. Given these findings, we then investigated the expression of miRNAs in the MS animal model, EAE at different phases of disease. EAE was induced in 30 animals which were divided into three groups for tissue extraction at three time points after the induction of disease. The first time point was day 10 post-induction before the development of any neurological signs (pre-onset); the second time-point was at the peak of the disease that varied between days 18 and 20 for mice in the group (“peak of disease phase”); and the third time point was at day 25 post-induction (“post peak phase”) (Fig. 1b). Immunohistochemical analysis of lumbar spinal cord tissue isolated from mice at the peak of disease showed infiltration of CD3 immunopositive T cells as well as reduced staining for myelin basic protein in EAE mice compared with CFA control animals (Additional file 1: Figure S2). Expression analysis for two miR-142 mature isoforms on the RNA extracted from spinal cord tissue showed substantial upregulation of miR-142a-5p and miR-142a-3p in the lumbar spinal cord in peak of disease and post peak phases of EAE compared with control mice (Fig. 1c).

### miR-142a-3p and miR-142a-5p are induced in activated splenocytes

**Fig. 2:**
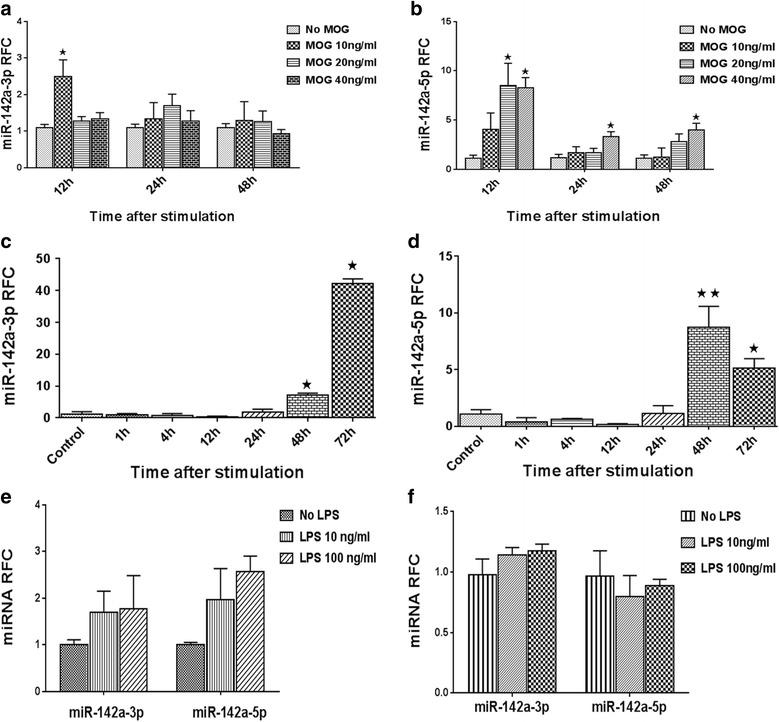
miR-142a-3p and miR-142a-5p expression levels in splenocytes, macrophages, and astrocytes. Expression levels of miR-142a-3p and miR-142a-5p were analyzed in MOG-treated splenocytes from immunized animals (**a**, **b**) or splenocytes stimulated with anti-CD3 and anti-CD28 for different time points (**c**, **d**). Bone marrow-derived macrophages (**e**) and primary astrocytes (**f**) were stimulated with two concentrations of LPS for 12 h, before expression analysis by RT-PCR. Data are shown as mean ± SEM, *n* = 3. Experiment was repeated twice. *p < 0.05, **p < 0.01, Kruskal–Wallis test

Different cell types are involved in the neuroinflammatory process in MS/EAE, with autoreactive T cells and monocytoid cells, i.e., infiltrating monocytes and locally activated microglia, being key players. Astrocytes have also been implicated in disease process by producing various inflammatory mediators. To examine the potential contribution of these cells to miR-142a upregulation during inflammation, different in vitro systems were used. First, the expression of miR-142a isoforms were analyzed in splenocytes isolated from mice immunized with MOG35–55. Cells were restimulated in culture with three concentrations of MOG peptide for different time points before lysis and RNA extraction. RT-PCR data showed that miR-142a-5p levels were significantly increased in MOG-treated splenocytes after 12 h at 20 and 40 ng/ml and after 24 and 48 h at 40 ng/ml concentrations of peptide. A significant upregulation was also detected for miR-142a-3p at 10 ng/ml concentration of peptide at 12 h of treatment (Fig. 2a, b). We next studied the expression of miRNAs in T cells polyclonally activated by anti-CD3 and anti-CD28 antibodies. To this end, RNA was extracted from the cells at a range of time points after activation. The analyses revealed a substantial increase in miR-142a-3p and miR-142a-5p levels in stimulated splenocytes after 48 and 72 h (Fig. 2c, d). Indeed, the miRNA upregulation was much stronger in anti-CD3/CD28-stimulated T cells compared with MOG-stimulated cells, an effect that was expected considering that only a small fraction of splenocytes in MOG-immunized animals is MOG-reactive. Primary cultures of bone marrow-derived macrophages (BMDM) and astrocytes were then established to study miRNA expression in these cell types. LPS stimulation of cells was used as a model that could recapitulate some of the features of monocyte/glial cell activation during disease [30, 31]. A mild increase in miR-142a-3p and miR-142a-5p levels was seen in LPS-stimulated macrophages, but it did not reach statistical significance (Fig. 2e). Likewise, activated primary astrocytes did not show any change in miR-142a-3p or miR-142a-5p levels following stimulation with LPS (Fig. 2f). Overall, these data indicate that splenocytes are able to enhance their expression of miR-142 isoforms after antigen-specific or polyclonal activation, and hence, they might be responsible for higher levels of miRNA transcripts identified in MS and EAE CNS tissues.

### miR-142a-5p induces differentiation of naïve CD4^+^ T cells towards Th1 subset

**Fig. 3:**
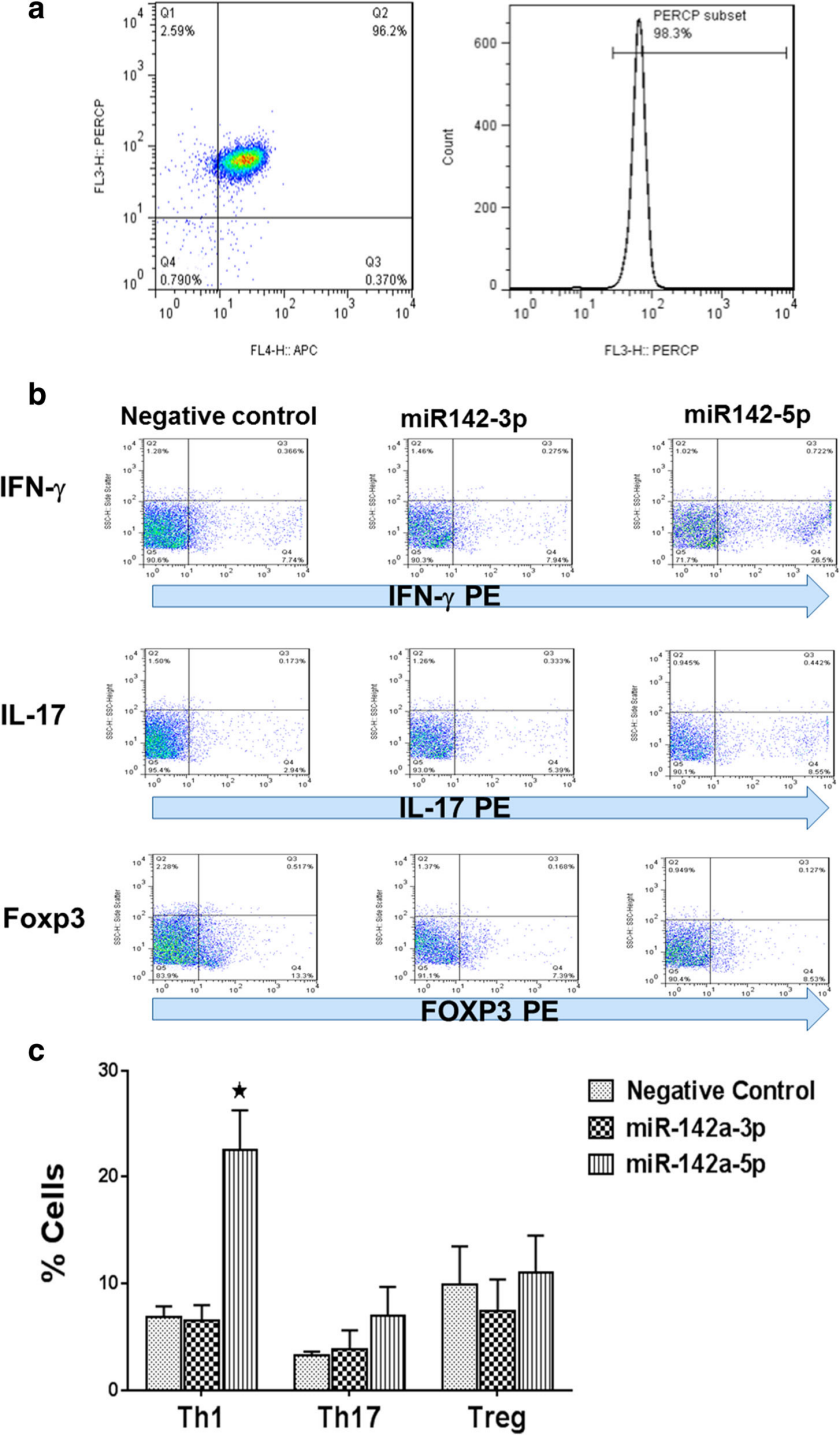
Overexpression of miR-142a-5p affects the differentiation of CD4^+^ T cells. Naïve CD4^+^ T cells were isolated from mouse splenocytes to a purity of approximately 95% (**a**). miR-142a-3p, miR-142a-5p, and negative control sequences were transfected into CD4^+^ T cells which were activated and polarized under relevant cytokine regimens. The frequencies of Th1, Th17, and Treg cells in CD4^+^ T cells were determined by intracellular staining and flow cytometry after 4 days. Representative dot plots and the percentages of IFN-γ, IL-17, and FoxP3 immunopositive cells within the CD4^+^ T cells (**b**). Average cell frequencies (**c**). Data are shown as mean ± SEM, *n* = 3. Data are from a single experiment representative of three independent experiments.(*p < 0.05, one-way ANOVA)

Considering the upregulation of miR-142 isoforms in antigen-stimulated or anti-CD3/CD28 activated splenocytes, we next investigated the effect of miR-142a isoforms on CD4 T cell differentiation. Naïve CD4^+^ T cells were purified from the splenocytes of C57/BL6 mice, with a purity of more than 95% (Fig. 3a). Purified CD4^+^ cells were transfected with miR-142a-3p and miR-142a-5p mimics and were then cultured in the presence of anti-CD3 and anti-CD28 in the presence of the required cytokines for differentiation to Th1, Th17, and Treg cells, as described in the “Methods” section. Intracellular cytokine staining and flow cytometric analysis after 4 days showed increased differentiation of miR-142a-5p-transfected cells towards IFN-γ producing Th1 subtype, compared with T cells transfected with a control miRNA sequence (Fig. 3b). There was also a mild increase in the frequency of IL-17-producing Th17 cells, but the increase did not reach statistical significance (Fig. 3c). The frequency of Treg cells did not reveal any difference following miR-142a-5p transfection. Similar experiments were performed for miR-142a-3p isoform. In contrast to miR-142a-5p, miR-142a-3p-transfected cells did not show any shifts in their differentiation towards Th1, Th17, or T regulatory cells, compared with T cells transfected with a scrambled miRNA control sequence. These data indicate that miR-142 isoforms can be upregulated in T cells following exposure to the antigen and activation of TCR signaling and their upregulation, at least for the 5p isoform, might be able to influence CD4 T cell status towards the more pathogenic Th1 phenotype (Fig. 3c).

### Expression of inflammation-related transcripts are regulated by miR-142 isoforms

miRNAs exert their effects by targeting protein-coding transcripts. To investigate the potential mRNA transcripts which might be targeted by each of miR-142 isoforms, we extracted a list of predicted mRNA targets from TargetScan and miRDB databases. Considering the high number of potential targets, we focused on targets with known roles in cytokine signaling which also show conserved miRNA binding sites between human and mouse. We selected TGFBR-1 as a predicted target for miR-142a-3p and TGFBR-2 and SOCS-1 as predicted targets for mir-142a-5p. Adenylate cyclase 9 (ADCY9) was also selected as a confirmed target for miR-142a-5p (Additional file 1: Table S2). In addition to the mature miRNA sequences, the mRNA binding sites on the 3′ UTR of these genes (TGFBR1, ADCY9, TGFBR2, and SOCS1) are conserved between human and mouse (Additional file 1: Figure S1).

**Fig. 4:**
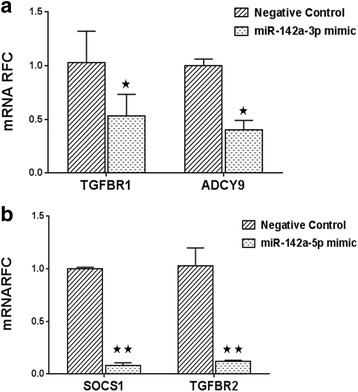
Overexpression of miRNA sequences affects target gene expression in stimulated splenocytes. The expression of potential target genes was examined in cells transfected with miRNA sequences by real-time RT-PCR. TGFBR1 and ADCY9 transcripts were significantly suppressed in cells overexpressing miR-142a-3p compared with miRNA negative control transfected cells (**a**). SOCS1 and TGFBR2 transcripts were also significantly suppressed in cells overexpressing miR-142a-5p compared with miRNA negative control overexpressing cells (**b**). Data are shown as mean ± SEM, *n* = 3. Experiment was repeated twice. **p < 0.01, *p < 0.05, Student’s *t* test

We first transfected miR-142a mimic sequences into mouse splenocytes and measured the target expression levels by real-time RT-PCR. TGFBR1 and ADCY9 transcript levels showed a significant reduction in miR-142a-3p-transfected cells in comparison with CD4^+^ T cells transfected with a negative control sequence (Fig. 4a). Likewise, in cells transfected with miR-142a-5p sequences the levels of TGFBR2 and SOCS1 were decreased significantly in comparison with negative control transfected cells (Fig. 4b).

### Expression of miR-142a isoform targets is dysregulated in activated splenocytes

**Fig. 5:**
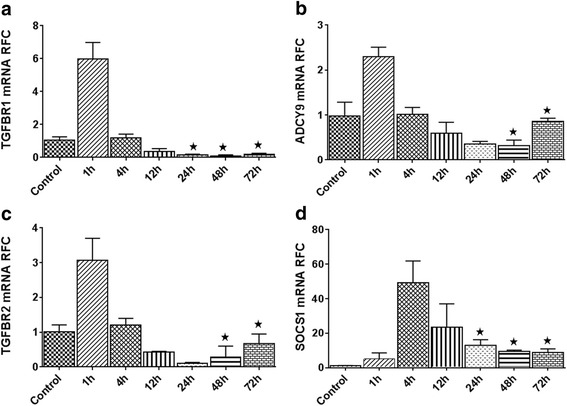
miR-142a-3p and miR-142a-5p predicted target genes expression in activated splenocytes. Expression of miR-142 isoforms target genes in stimulated splenocytes was determined by real-time RT-PCR. The levels of TGFBR1 and ADCY9 genes in 24, 48, and 72 h (**a**, **b**). TGFBR2 in 24 and 48 h after stimulation were significantly decreased in stimulated splenocytes compared with unstimulated cells (**c**). SOCS1 levels were initially upregulated followed by a significant reduction (**d**). Data are shown as mean ± SEM, *n* = 3. Experiment was repeated twice. *p < 0.05, one-way ANOVA)

As shown in Fig. 2, the expression of miR-142a isoforms increased in stimulated splenocytes after 48 and 72 h. Hence, to investigate whether altered miRNA expression is associated with any changes in the expression levels of potential targets, we analyzed the expression levels of miR-142a-3p predicted targets, TGFBR1, and ADCY9, as well as miR-142a-5p predicted targets, TGFBR2, and SOCS1 in stimulated splenocytes. TGFBR1 mRNA levels showed an initial upregulation after 1 h of stimulation compared with untreated cells; however, the expression quickly decreased in subsequent time points, to levels significantly below control cells after 24 h of stimulation (Fig. 5a). ADCY9 levels also displayed a similar downregulation after 24 h (Fig. 5b). TGFBR2 and SOCS1 showed an initial induction but were substantially downregulated at time points after 24 h (Fig. 5c, d). Overall, these data show changes in expression of the targets which are “anti-parallel” with miRNA levels in the cells, further supporting their potential regulation by miRNAs.

### Luciferase assays show direct interaction between miR-142a isoforms and mRNA transcripts

**Fig. 6:**
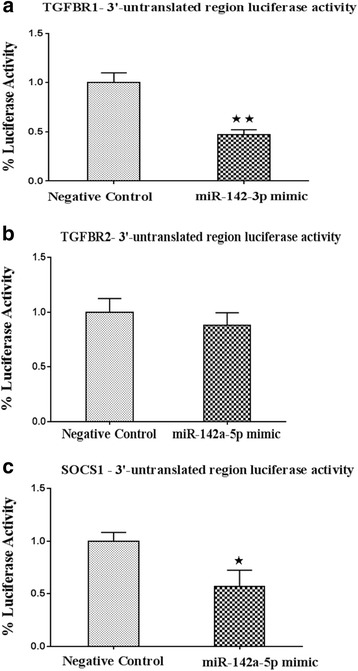
Luciferase-3′-untranslated region reporter assay. Interaction of miR-142a-3p with TGFBR1 and miR-142a-5p with TGFBR2 and SOCS1 transcripts were assessed using luciferase assay system. Co-transfection of HEK293T cells containing luciferase-3′UTR construct from TGFBR1 together with miR-142a-3p mimics showed significant suppression of luciferase activity in comparison with cells transfected with a control miRNA sequence (**a**). miR-142a-5p transfection did not lead to significant suppression of luciferase activity in cells co-transfected with TGFBR2 3′UTR constructs (**b**). Similar experiments with luciferase-3′UTR from SOCS1 and miR-142a-5p mimics showed suppression of luciferase activity compared with cells transfected a control sequence (**c**). Firefly luciferase levels were normalized against renilla luciferase, expressed as an internal control in the vector. Data are shown as mean ± SEM, *n* = 5. Data are from a single experiment representative of three independent experiments. *p < 0.05, **p < 0.01, Student’s *t* test

To examine whether miR-142a directly interacts with TGFBR1, TGFBR2, and SOCS1 transcripts, we used a luciferase reporter system. The 3′-UTR region of TGFBR1 mRNA was PCR amplified and cloned into psiCHECK-2 vector, downstream of firefly luciferase coding sequence. HEK293T cells were co-transfected with luciferase-3′UTR construct and miR-142a-3p mimics or a negative miRNA control. Quantification of luciferase activity after co-transfection revealed significant suppression of luciferase activity in cells co-transfected with miR-142a-3p mimics in comparison with cells transfected with the negative control miRNA (Fig. 6a). Similar experiments were performed for miR-142a-5p. The 3′-UTR region of TGFBR2 and SOCS1 mRNAs were cloned into psiCHECK-2 vector, separately. HEK293T cells were then co-transfected with vectors encoding the firefly luciferase open reading frame fused to the 3′-UTR region of TGFBR2 or SOCS1 and mimics of miR-142a-5p or negative control miRNA. Quantification of luciferase activity after co-transfection revealed significant reduction in luciferase activity in cells co-transfected with SOCS1-3′-UTR region and miR-142a-5p in comparison with cells transfected with a negative control miRNA (Fig. 6c). Nonetheless, in cells co-transfected with miR-142a-5p mimics and TGFBR2-3′-UTR constructs, luciferase activity did not show any significant suppression (Fig. 6b). Altogether, these data indicate that miR-142a-3p and miR-142a-5p isoforms directly target TGFBR1 and SOCS1 transcripts, respectively. In the case of TGFBR2 our data did not reveal evidence of direct interactions, however, considering the results of T cells transfection experiments, it seems that miR-142-3p might be able to affect TGFBR2 levels, perhaps by indirect means.

### miR-142a isoforms target genes show dysregulation in the CNS of patients with multiple sclerosis and animals with EAE

**Fig. 7:**
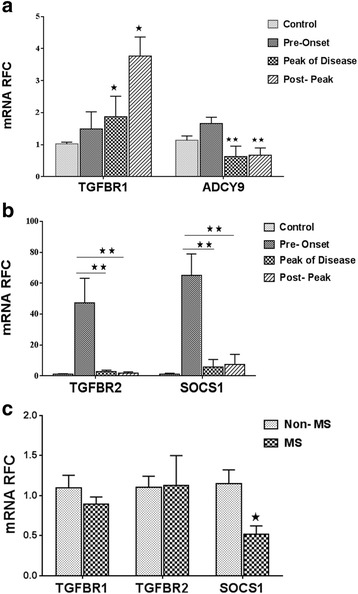
miR-142a-3p and miR-142a-5p target gene expression in EAE tissue and human MS brain samples. miR-142a-3p target gene, TGFBR1, was significantly increased in peak of disease and post peak phase of EAE in spinal cord (**a**). SOCS1 and TGFBR2 were significantly decreased in peak of disease and post peak phases of EAE in comparison with pre-onset phase (**b**). In human autopsy samples, SOCS1 showed significant reduction in MS samples but the levels of TGFBR1 and TGFBR2 did not differ between MS and control tissues (**c**). Data are shown as mean ± SEM, **p < 0.01, *p < 0.05, one-way ANOVA

Given the increased expression of miR-142 isoforms in human and miR-142a isoforms in EAE CNS tissues we next investigated the expression levels of each target transcript in MS and EAE tissues. We analyzed the transcript levels of TGFBR1 and ADCY9 as targets of miR-142a-3p, as well as SOCS1 as a target of miR-142a-5p in the human brain samples and in the lumbar spinal cord tissue from EAE mice in three phases of disease (pre-onset, peak of disease and post peak) using real-time PCR. Data in EAE tissue showed that the expression of TGFBR1 was increased in peak of disease and post peak phases of disease (Fig. 7a), whereas the expression levels of ADCY9 was lower in peak of disease and post peak stages of disease compared with controls or the pre-onset phase (Fig. 7a). SOCS1 expression showed a significant upregulation before the onset of neurological signs but it was downregulated at later stages of the disease (Fig. 7b). Expression of TGFBR2, an indirect target of miR-142a-5p also showed a similar pattern (Fig. 7b). Analysis of the human white matter showed significant reduction in SOCS1 transcript levels in comparison with non-MS samples but TGFBR1 and TGFBR2 expression levels were similar between patient groups with a trend towards reduced TGFBR1 expression in MS white matter (Fig. 7c).

## Discussion

In this study, we investigated the role of miR-142a isoforms (miR-142a-3p and miR-142a-5p) in autoimmune neuroinflammation. Using human brain autopsy samples and EAE CNS tissues, as well as different cell culture systems and molecular analyses, we show that miR-142 isoforms might be involved in the neuroinflammatory processes underlying MS/EAE. Our gene expression studies showed increased levels of both miR-142-5p and miR-142-3p isoforms in EAE spinal cord, and in MS brains miR-142-5p showed statistically significant increase. Consistent with previous reports [32], our in vitro gene expression studies showed expression of miR-142 mature isoforms in both T cells and primary macrophages. Both cell types contribute to neuroinflammation in MS/EAE. MS tissues used in this study were derived from the so-called normal-appearing white matter (NAWM) around lesions. It should be noted that while inflammation is most severe in classical MS lesions, studies on NAWM have shown diffuse axonal injury together with microglial activation and T cell infiltration in these areas [33–35]. Of note, T cell receptor expression analysis in different brain regions has shown that the same T cell clones that are present in lesions are also present in NAWM indicating that similar antigens are recognized by T cells in lesions and NAWM [36]. While the current study is chiefly focused on miR-142 expression in T cells, we believe that both T cells and activated microglia in the NAWM contribute to enhanced miRNA expression in the tissues from MS patients.

Some very recent studies have pointed to the role of miR-142 isoforms in MS pathogenesis. Mandolesi et al. have reported increased levels of miR-142-3p in the CSF of patients with active MS as well as brain tissue from EAE mice [21]. miR-142-3p was shown to regulate IL-1beta-dependent synaptic abnormalities that occur during neuroinflammation. Interestingly, inhibition of miR-142-3p prevented an increase in glutamergic transmission caused by exposure of cerebellar slices to CSF from MS patients [21]. Studies on blood cells have also linked miR-142-3p with MS disease process. Arruda et al. have recently reported enhanced miR-142-3p expression in CD4^+^ and CD8^+^ T cells from MS patients [37]. Conversely, some T cell studies have reported downregulation of miR-142-3p. In a study by Sanders et al., researchers performed next generation sequencing on CD4^+^ T cells from secondary progressive multiple sclerosis (SP-MS) patients. The results revealed downregulation of multiple miRNAs including miR-142-3p in T cells derived from MS patients [38]. These discrepancies could likely be a reflection of disease heterogeneity as well as highly dynamic nature of miRNA expression during different phases of disease.

Our data suggest that the miR-142 isoforms could target transcripts which are involved in cytokine signaling and T cell differentiation, thereby affecting the phenotype of neuroantigen-reactive T cells infiltrating the nervous system during disease. We show that “suppressor of cytokine signaling 1”, SOCS1, is a direct target of miR-142a-5p. SOCS1 is a member of the SOCS family of proteins which are negative regulators of cytokine signaling. Multiple cytokines recruit the Janus kinase (JAK)–signal transducers and activators of the transcription (STAT) molecules to exert their effects [39, 40]. The proteins of SOCS family which are induced by different cytokines including IL-2 and IFN-γ can impede the signal transduction by these and other cytokines through inhibition of JAKs or blockade of their recruitment to the cytokine receptors [41]. SOCS1, one the most widely studied members of the SOCS family, has been shown to be involved in regulating the differentiation of T cells, making the molecule a key player in T cell-mediated immunopathologies [42]. It is known that STAT1 and STAT5 contribute to Th1 differentiation by enhancing T-bet and IFN-γ expression [43, 44]. SOCS1 suppresses STAT1 [41] and blocks IFN-γ-mediated STAT1 activation by targeting JAK2 and IFN-cRa chain [45]. In addition to its role in Th1 development, SOCS1 is necessary for Treg stability and suppressor function through stabilizing Foxp3 expression [46] and by preventing the production of inflammatory cytokines by Tregs [47]. Normally, Tregs do not secret inflammatory cytokines but in the absence of SOCS1, these cells secret IFN-γ and IL-17 likely due to hyperactivation of STAT1 and STAT3 [48]. This phenomenon can lead to the loss of Foxp3 expression and the conversion of regulatory cells to a Th1/Th17 phenotype [49]. Indeed, studies of SOCS1 KO mice have shown that most SOCS1-deficient CD4 naïve T cells differentiate into Th1 [50, 51] and SOCS1-deficient mice develop autoimmune inflammatory diseases with age [31]. All these findings point to SOCS1 as a guardian of Tregs and a controller of Th1 development. In this study, we show that SOCS1 levels are diminished in MS tissues, and that it could be targeted by miR-142-5p, a finding that can be viewed important both from the perspectives of understanding MS pathogenesis and potential therapeutic interventions.

The other isoform of miR-142, i.e., miR-142-3p, has also been implicated in regulating T cell activity. Indeed, it has been reported that miR-142-3p reduces the production of cyclic 3′5′-adenosine monophosphate (cAMP), a molecule required for regulatory function of Tregs, by suppressing adenylyl cyclase (AC) 9 mRNA in T cells and macrophages. Apparently, miR-142-3p does not influence the expression of Foxp3, but Foxp3 regulates, directly or indirectly, the expression of miR-142-3p [19]. In this study, using overexpression experiments and luciferase assays, we showed that TGFBR1 is a target of miR-142a-3p. TGF-β is a cytokine with pleiotropic functions including regulation of inflammation as well as survival, growth and differentiation of many cell types [32]. TGF-β signaling is initiated by the binding of TGF-β to heteromeric complexes of type I (TGFbRI) and type II (TGFbRII) receptors on the cell membrane [52]. Several studies have shown the enhanced expression of TGF-β1 in the CNS in MS and EAE [53–57]. The effects of TGFβ1 in the context of MS/EAE are diverse and chiefly protective. These effects could be roughly categorized to two types: effects on neural cells and on leukocytes. TGF-β1’s promotion of oligodendrocyte differentiation leading to enhanced remyelination in MS lesions is an example of the effects on neural cells [58, 59]. TGFβ1 also suppresses autoantigen-induced activation of lymphocytes, activation of monocytoid cells, and the production of pro-inflammatory cytokines [60]. This latter function is believed to be chiefly mediated by increasing Treg activity. The finding that miR-142a-3p can target TGFBR1 and thereby diminish TGFβ signaling might point to a novel pathogenic pathway that diminishes both neuroprotective and immunomodulatory effects of the cytokine simultaneously. Indeed, it has been demonstrated that the expression of miR-142a-3p in Treg cells is lower compared with non Treg CD4^+^ T cells. While we did not observe a shift in T cell differentiation following miR-142a-3p transfection of these cells, the possibility still remains that miRNA-mediated reduction in TGFBR1 expression in T cells, monocytoid cells, or neural cells might play a role in neuroinflammation/degeneration.

## Conclusions

Results of this study suggest that miR-142 isoforms are upregulated in the CNS of MS patients and animals with EAE. Increased expression of these microRNAs might be involved in the autoimmune neuroinflammation and pathogenesis of multiple sclerosis through changing the pattern of T cells differentiation towards IFN-γ-producing Th1 cells; an effect which might be mediated through targeting and suppression of protective genes such as TGFBR1 and SOCS1.

## Additional file

Additional file 1: Figure S1. miR-142 isoforms homology between human and mouse (a). miR-142 isoforms binding region in human and mouse for ADCY9 (b), TGFBR1 (c), TGFBR2, and SOCS1 (d). **Figure S2.** Validation of the EAE model by immunohistochemistry with anti-CD3 and anti-MBP antibodies. **Table S1.** Primer sets used in this study. **Table S2.** TargetScan and miRDB predicted targets for miR-142-3p and miR-142-5p. miRTarbase sequences are experimentally confirmed human miRNA-mRNA interactions. (PPTX 2023 kb)

## Abbreviations

ADCY9: Adenylate cyclase 9
BMDM: Bone marrow-derived macrophages
cAMP: Cyclic adenosine monophosphate
CNS: Central nervous system
EAE: Experimental autoimmune encephalomyelitis
IFN-γ: Interferon gamma
JAK: Janus kinase
LPS: Lipopolysaccharide
M-CSF: Macrophage colony-stimulating factor
MOG: Myelin oligodendrocyte glycoprotein
MS: Multiple sclerosis
RR-MS: Relapsing-remitting multiple sclerosis
RT-PCR: Reverse transcriptase polymerase chain reaction
SOCS1: Suppressor of cytokine signaling 1
SP-MS: Secondary progressive multiple sclerosis
STAT1: Signal transducer and activator of transcription 1
TGFBR: Transforming growth factor beta receptor
TGF-β: Transforming growth factor
Th cell: T helper cell
Treg cell: Regulatory T cell
