# Supplementary material for: MicroRNA-142 regulates inflammation and T cell differentiation in an animal model of multiple sclerosis
Source: J Neuroinflammation. 2017 Mar 16;14:55. doi: 10.1186/s12974-017-0832-7 (PMC5356264; doi:10.1186/s12974-017-0832-7)
Supplement: Additional file 1: Figure S1. — miR-142 isoforms homology between human and mouse (a). miR-142 isoforms binding region in human and mouse for ADCY9 (b), TGFBR1 (c), TGFBR2, and SOCS1 (d). Figure S2. Validation of the EAE model by immunohistochemistry with anti-CD3 and anti-MBP antibodies. Table S1. Primer sets used in this study. Table S2. TargetScan and miRDB predicted targets for miR-142-3p and miR-142-5p. miRTarbase sequences are experimentally confirmed human miRNA-mRNA interactions. (PPTX 2023 kb) [file 12974_2017_832_MOESM1_ESM.pptx]

## Slide 1
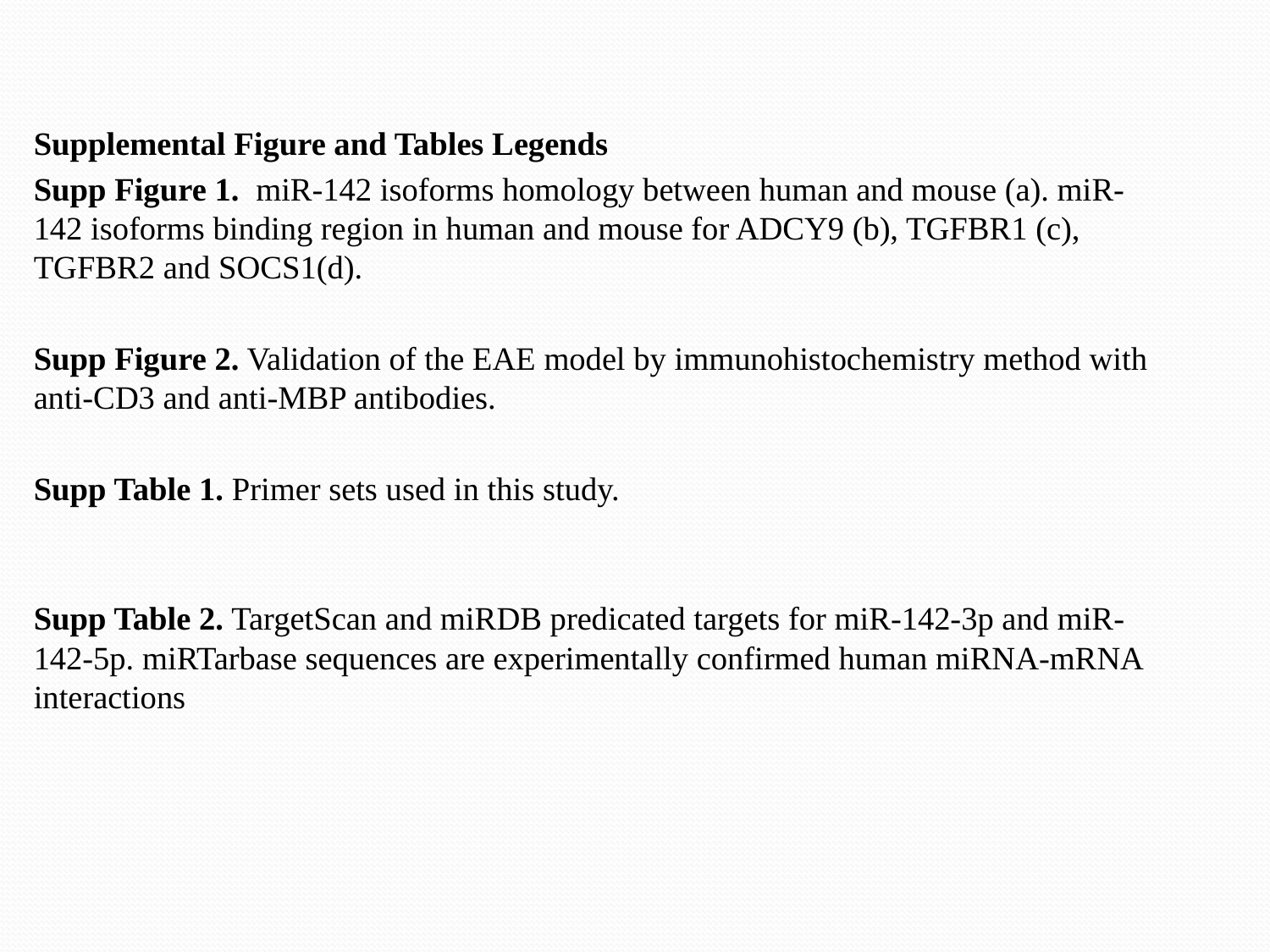

Supplemental Figure and Tables Legends
Supp Figure 1. miR-142 isoforms homology between human and mouse (a). miR-142 isoforms binding region in human and mouse for ADCY9 (b), TGFBR1 (c), TGFBR2 and SOCS1(d).
Supp Figure 2. Validation of the EAE model by immunohistochemistry method with anti-CD3 and anti-MBP antibodies.
Supp Table 1. Primer sets used in this study.
Supp Table 2. TargetScan and miRDB predicated targets for miR-142-3p and miR-142-5p. miRTarbase sequences are experimentally confirmed human miRNA-mRNA interactions

## Slide 2
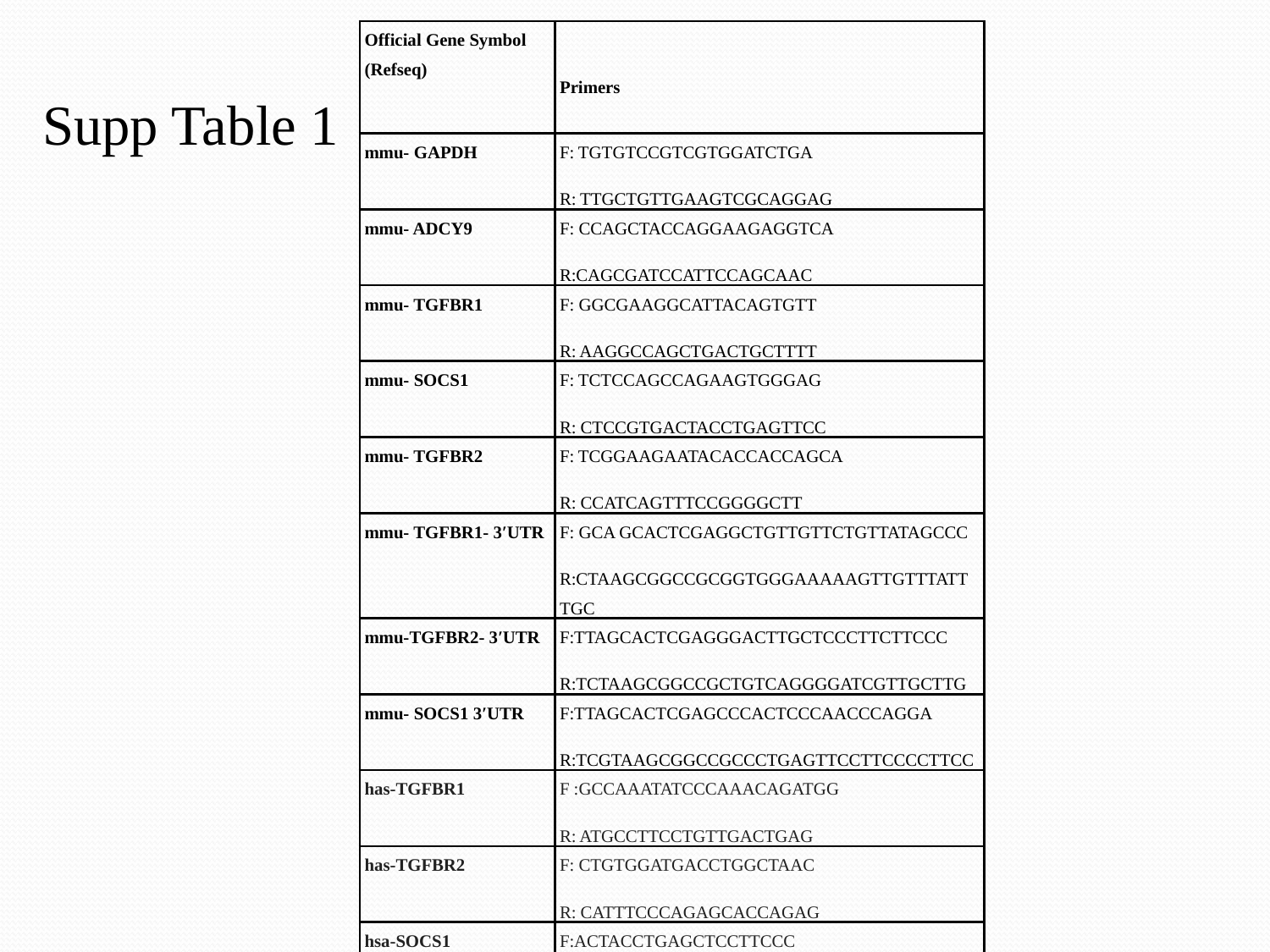

# Supp Table 1
| Official Gene Symbol (Refseq) | Primers |
| --- | --- |
| mmu- GAPDH | F: TGTGTCCGTCGTGGATCTGA R: TTGCTGTTGAAGTCGCAGGAG |
| mmu- ADCY9 | F: CCAGCTACCAGGAAGAGGTCA R:CAGCGATCCATTCCAGCAAC |
| mmu- TGFBR1 | F: GGCGAAGGCATTACAGTGTT R: AAGGCCAGCTGACTGCTTTT |
| mmu- SOCS1 | F: TCTCCAGCCAGAAGTGGGAG R: CTCCGTGACTACCTGAGTTCC |
| mmu- TGFBR2 | F: TCGGAAGAATACACCACCAGCA R: CCATCAGTTTCCGGGGCTT |
| mmu- TGFBR1- 3′UTR | F: GCA GCACTCGAGGCTGTTGTTCTGTTATAGCCC R:CTAAGCGGCCGCGGTGGGAAAAAGTTGTTTATTTGC |
| mmu-TGFBR2- 3′UTR | F:TTAGCACTCGAGGGACTTGCTCCCTTCTTCCC R:TCTAAGCGGCCGCTGTCAGGGGATCGTTGCTTG |
| mmu- SOCS1 3′UTR | F:TTAGCACTCGAGCCCACTCCCAACCCAGGA R:TCGTAAGCGGCCGCCCTGAGTTCCTTCCCCTTCC |
| has-TGFBR1 | F :GCCAAATATCCCAAACAGATGG R: ATGCCTTCCTGTTGACTGAG |
| has-TGFBR2 | F: CTGTGGATGACCTGGCTAAC R: CATTTCCCAGAGCACCAGAG |
| hsa-SOCS1 | F:ACTACCTGAGCTCCTTCCC R :CAAAATAACACGGCATCCCAG |

## Slide 3
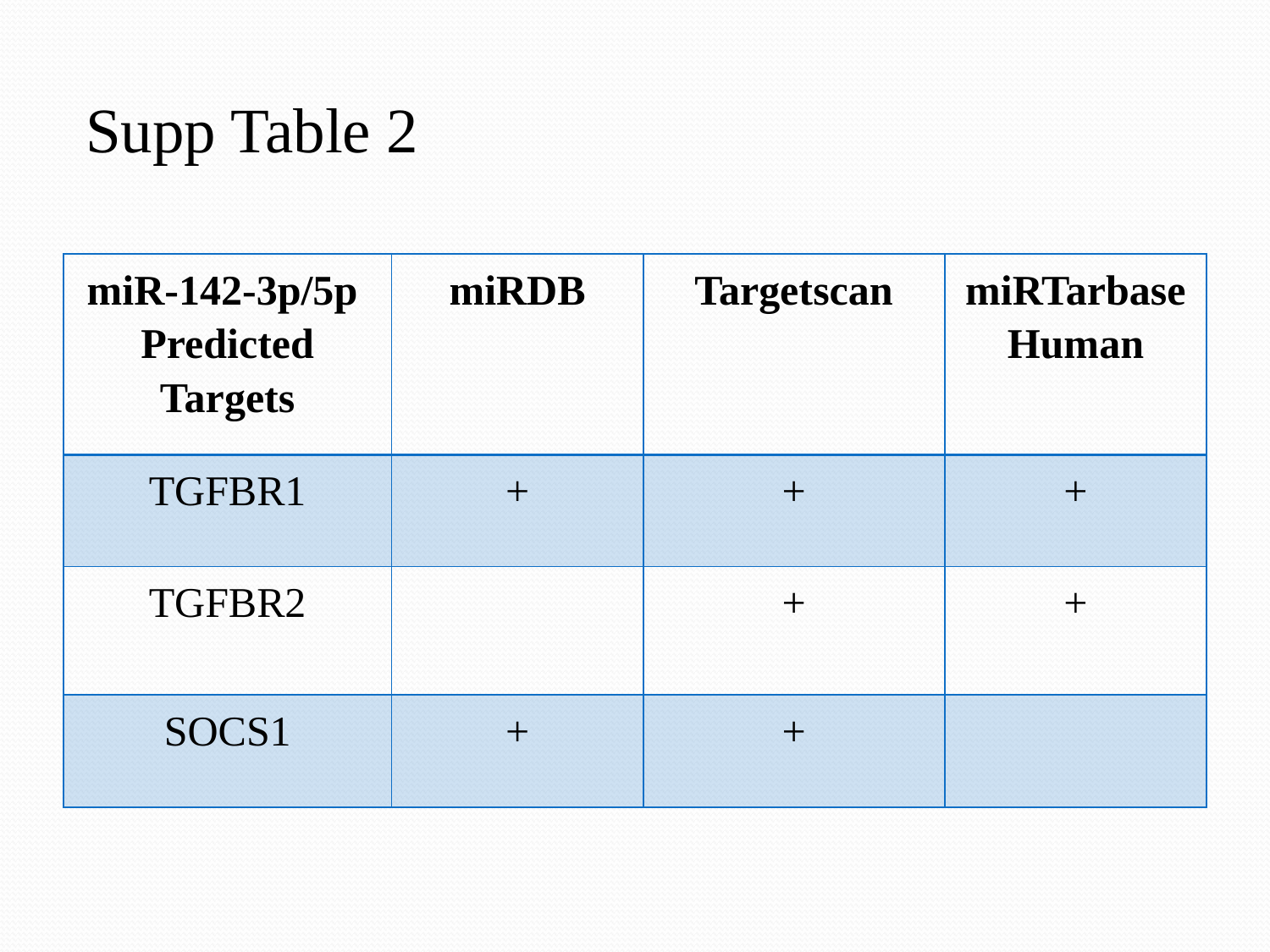

Supp Table 2
| miR-142-3p/5p Predicted Targets | miRDB | Targetscan | miRTarbase Human |
| --- | --- | --- | --- |
| TGFBR1 | + | + | + |
| TGFBR2 | | + | + |
| SOCS1 | + | + | |

## Slide 4
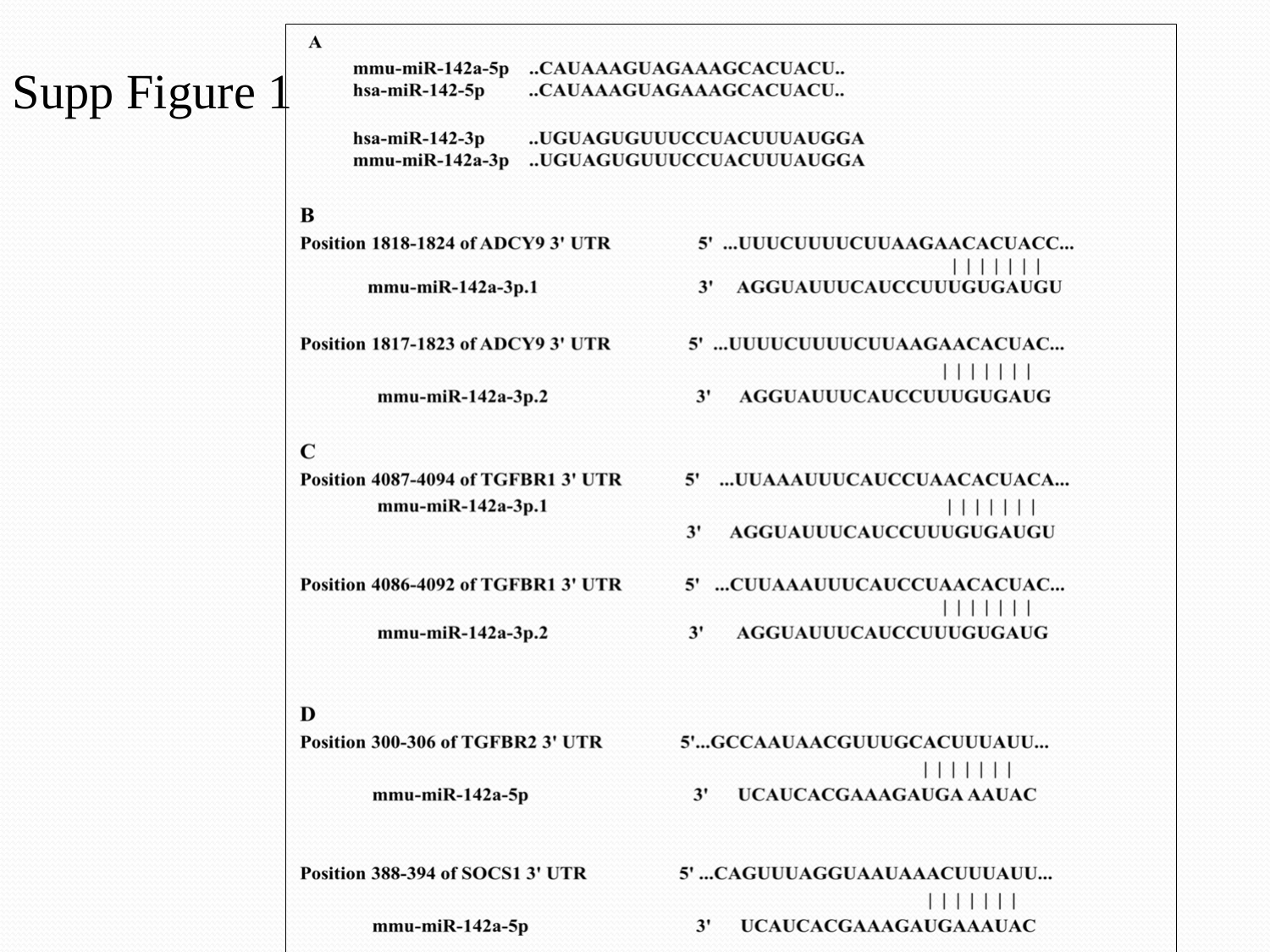

Supp Figure 1

## Slide 5
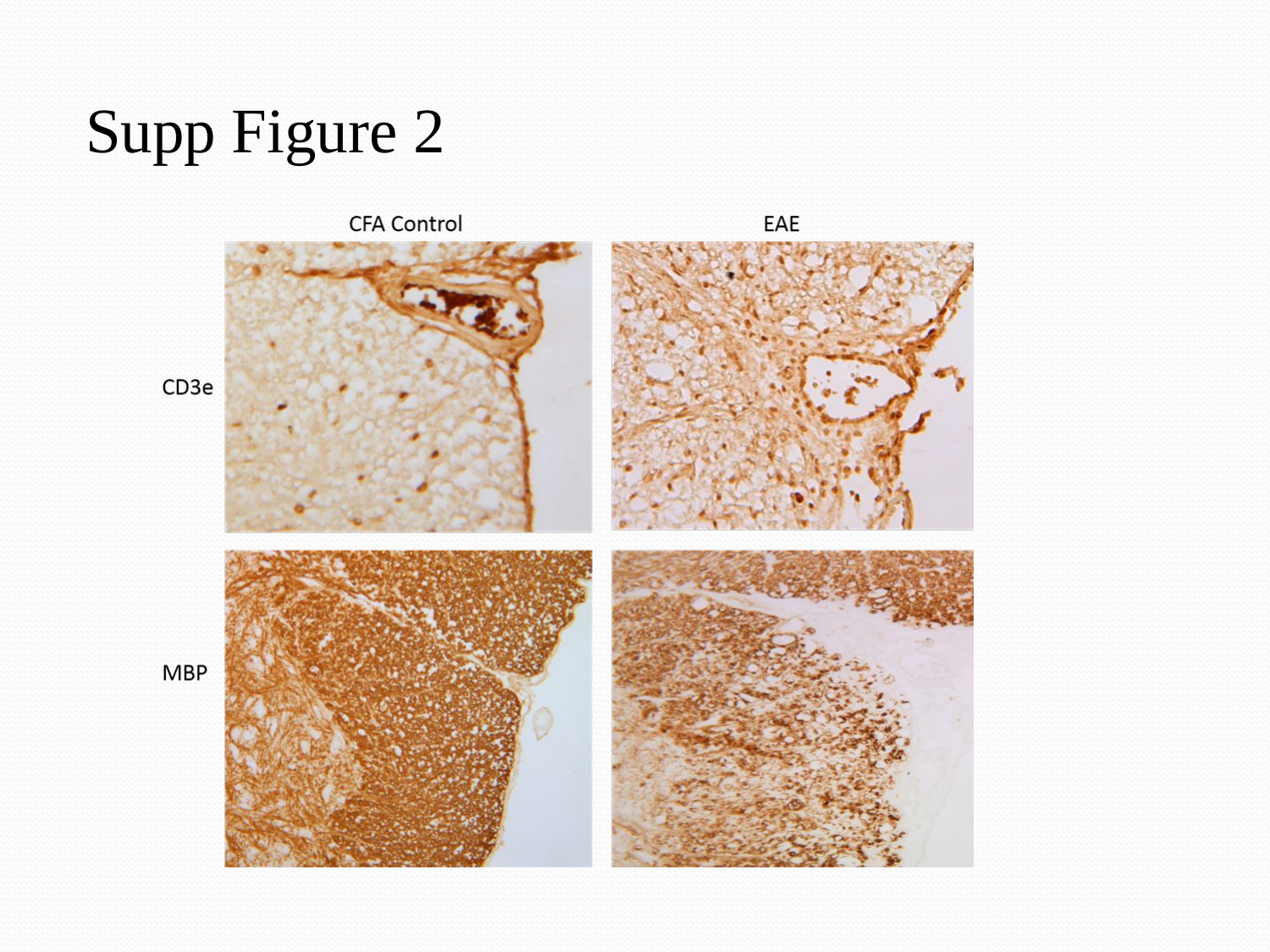

Supp Figure 2
